# Chloroplast genome sequencing and divergence analysis of 18 *Pyrus* species: insights into intron length polymorphisms and evolutionary processes

**DOI:** 10.3389/fgene.2024.1468596

**Published:** 2024-10-23

**Authors:** Jung Sun Kim, Hoyong Chung, Bohyeon Park, Karpagam Veerappan, Yoon-Kyung Kim

**Affiliations:** ^1^ Genomics Division, National Institute of Agricultural Sciences, Rural Development Administration, Jeonju, Republic of Korea; ^2^ 3BIGS Co., Ltd., Suwon, Republic of Korea; ^3^ Pear Research Institute, National Institute of Horticultural and Herbal Science, Rural Development Administration, Naju, Republic of Korea

**Keywords:** chloroplast genome, *Pyrus ussuriensis*, *Pyrus pyrifolia*, phylogeny, next-generation sequencing

## Abstract

Pears constitute an essential temperate crop and are primarily produced through interspecific hybridization owing to self-incompatibility that complicates their breeding history. To address this, we sequenced the complete chloroplast (cp) genomes of 18 *Pyrus* and one *Malu*s species using the Illumina HiSeq4000 platform. The cp genomes ranged from 159,885 bp to 160,153 bp and exhibited a conserved circular DNA structure with an average GC content of 36.5%. Each cp genome contained 127 genes, including 83 protein-coding, 36 tRNA, and 8 rRNA genes. Divergence analysis with mVISTA showed high conservation in the coding regions and notable variations in the non-coding regions. All species shared 17 intron-containing genes, with *ycf3* and *clpP* each having two introns. Five intron-containing genes (*ndhB*, *rpl2*, *rps12*, *trnA-UGC*, and *trnE-UUC*) were located in the inverted repeat regions, while *trnL-UAA* was located in the large single-copy region, with conserved intron lengths across Pomoideae. We identified polymorphic intron sequences in the *rpl22*, *petB*, *clpP*, *ndhA*, and *rps16* genes and designed primers for these regions. Notably, the two *Pyrus ussuriensis* accessions Doonggeullebae and Cheongdangrori showed intron-length polymorphisms despite being classified as the same species. Phylogenetic analysis of the cp genome sequences revealed two major clusters, indicating distinct maternal lineages and evolutionary origins. This study underscores the importance of cp gene polymorphisms in *P. fauriei*, *P. calleryana*, *P. ussuriensis*, and *P. pyrifolia*, providing valuable insights into *Pyrus* evolution as well as aiding in the conservation and breeding of pear germplasm.

## 1 Introduction

Pears (*Pyrus*) are among the most highly valued fruit crops within the Rosaceae family and are some of the oldest cultivated fruits, with a history exceeding 3000 years (Rubtsov, 1944). These fruits are widely grown across diverse geographical regions, including Europe, North America, Central Asia, Asia Minor, and East Asia. Based on their geographical distribution, pears are classified into occidental (European) and oriental (Asian) pears (Hedrick et al., 1924). With 22 known species and over 5,000 varieties, pears are distributed across temperate regions in Europe, Asia, and northern parts of the United States (Monte-Corvo et al., 2001). The most prominent European pear is *P. communis*, while the main varieties of Asian pears include *P. pyrifolia*, *P. bretschneideri*, *P. ussuriensis*, *P. calleryana*, and *P. fauriei*. European pears are typically bell-shaped with smooth flesh and a strong aroma, whereas Asian pears are generally round with crisp flesh, mild flavor, and high sugar content (Wu et al., 2018).

Owing to their self-incompatibility, pears are frequently products of natural and artificial interspecific hybridization that have resulted in extensive genetic variations (Sassa et al., 1992; Claessen et al., 2019). Modern breeding programs aim to meet the increasing demand for pears by improving the available varieties through genetic selection and hybridization. To achieve these goals, a thorough understanding of the genetic diversity and relationships among pear accessions is crucial.

Chloroplast (cp) are dynamic photosynthetic organelles in green plant cells and contain their own genetic systems; cp genomes are characterized by small sizes, high copy numbers, uniparental inheritance, and low rates of recombination and mutation, serving as valuable genetic resources for species identification and phylogenetic studies (Wicke et al., 2011). Additionally, the study by Jansen et al. (2007) demonstrated that cp genomes show fewer genetic changes over time compared to nuclear genomes (Jiang et al., 2023). The stability and slow evolution of cp DNA make it particularly useful for resolving complex phylogenetic relationships in angiosperms; cp genome sequencing also provides insights into genetic relationships and evolutionary history across various plant families (Graham and Olmstead, 2000; Li et al., 2024). Compared to nuclear genomes, cp genomes are smaller, less prone to recombination, and have lower nucleotide substitution rates, making them ideal for phylogenetic and evolutionary studies (Allen, 2015; Abreu et al., 2018).

One of the defining characteristics of cp genomes is their slow evolutionary change; this makes cp genomes particularly useful for studying long-term evolutionary relationships and tracing lineage-specific variations (Wicke et al., 2011). Despite their structural conservation, cp genomes can undergo rearrangements, including deletions and duplications, which can contribute to phylogenomic analyses and understanding of species evolution (Graham and Olmstead, 2000).

Pear breeding programs have been underway for over a century to enhance the pear varieties and ensure their market sustainability. Interspecific hybridization has resulted in improved varieties with superior fruit qualities. Various cp-based molecular markers have been developed to distinguish accessions and understand the genetic as well as phylogenetic relationships among wild and domesticated plants (Dong et al., 2012; Bi et al., 2018; Li et al., 2020). For instance, studies on the genetic diversity and parentage of pear accessions using cp markers have provided significant insights into their breeding history and evolutionary trajectories (Ferradini et al., 2017; Li et al., 2018). Similarly, research on cp genome comparisons among the pear varieties has elucidated the phylogenetic relationships and genetic diversity within the genus *Pyrus* (Yue et al., 2018; Chung et al., 2019).

Sequencing the cp genomes of the Asian pear species is pivotal for elucidating their genetic relationships and evolutionary history. Advances in next-generation sequencing (NGS) technologies have accelerated cp genome availability in land plants, including those in the Rosaceae family. Comparative analyses of cp genomes across the *Pyrus* species have revealed conserved regions and genetic variations essential to the phylogenetic studies and breeding programs (Ferradini et al., 2017; Li et al., 2018; Yue et al., 2018).

In this study, we sequenced and assembled the complete cp genomes of 18 *Pyrus* and one *Malus* species to investigate the evolutionary history and genetic diversity within *Pyrus*. We focused on the conserved coding sequences (CDSs) and identified several intron-containing genes (*clpP*, *ndhA*, *rps16*, *petB*, and *rpl22*) with notable polymorphisms. Remarkably, two *P. ussuriensis* accessions Doonggeullebae (DG) and Cheongdangrori (CDR) exhibited intron-length polymorphisms despite being classified within the same species. This research provides significant insights into the genetic relationships and evolutionary dynamics of the *Pyrus* species to facilitate their conservation and breeding. Additionally, this study contributes to broader understanding of cp genome evolution as well as its application in phylogenetic and genetic studies in temperate fruit crops.

## 2 Results

### 2.1 Chloroplast genome characterization

#### 2.1.1 General cp genome features

**FIGURE 1:**
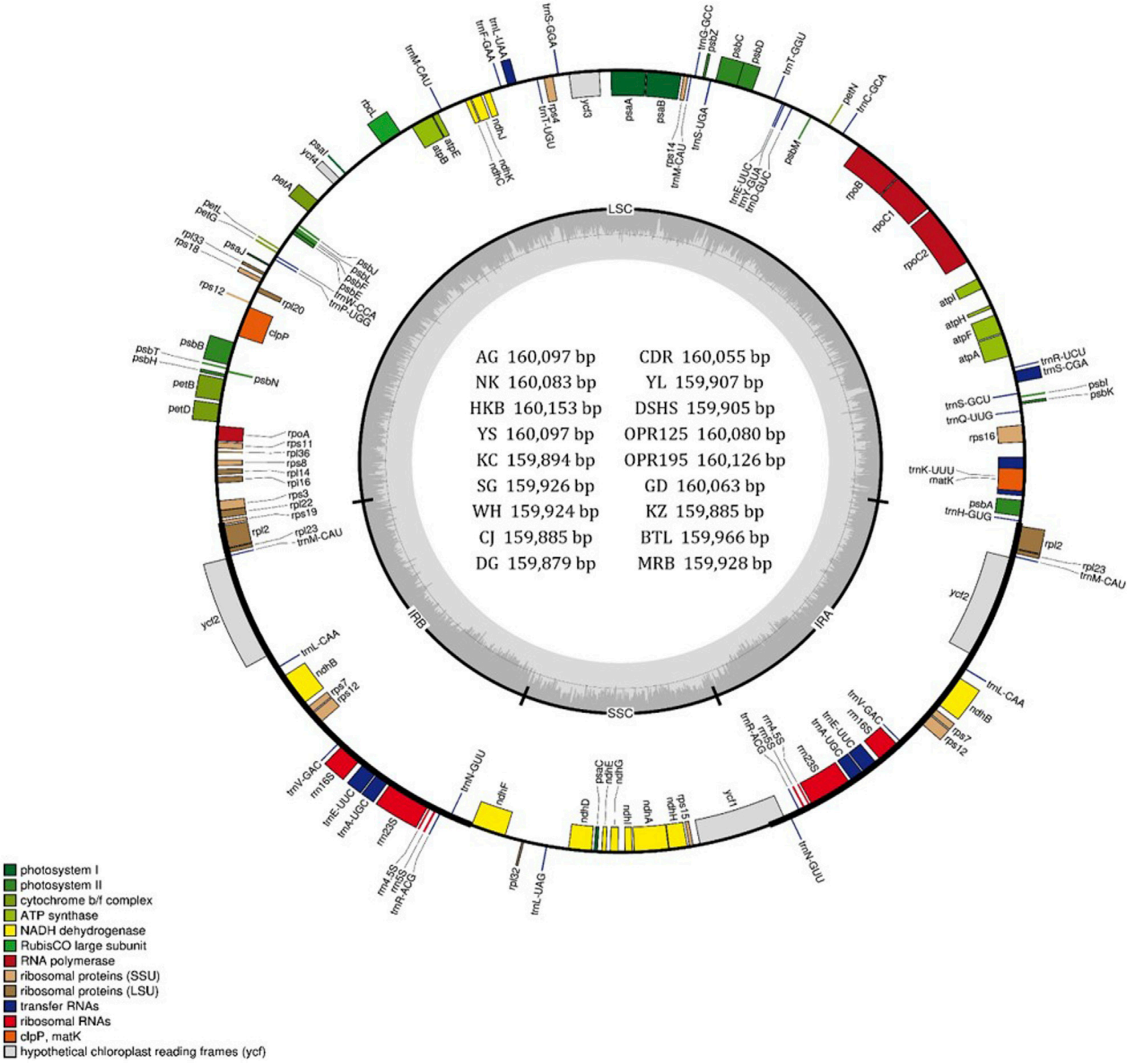
Gene map of the chloroplast (cp) genomes of 18 *Pyrus* species. The two thick lines indicate the extent of the inverted repeat (IRa and IRb) regions that divide the genome into the small single-copy (SSC) and large single-copy (LSC) regions. Genes on the outside of the circle are transcribed in the counterclockwise direction while those inside the circle are transcribed in the clockwise direction. Genes belonging to different functional groups are color-coded. The dark gray and light gray of the inner circle correspond to the GC and AT components, respectively. The map was drawn using OGDRAW. The cp genomes in the inner circle are Amanogawa (AG), Niitaka (NK), Whangkeumbae (HKB), Youngsanbae (YS), Imamuraaki (KC), SuperGold (SG), Wonwhang (WH), Chojuro (CJ), Doonggeullebae (DG), Cheongdangrori (CDR), Yali (YL), Dangshansuli (DSHS), Godang 5-1 (GD), Kozo (KZ), Bartlett (BTL), and Max Red Bartlett (MRB).

In this study, the cp genomes of 19 species (8 of *P. pyrifolia*, 2 of *P. ussuriensis*, 2 of *P. bretschneideri*, 2 of *P. calleryana*, 2 of *P. communis*, 1 of *P. fauriei*, 1 unknown, and 1 of *M. domestica*) were sequenced using the Illumina platform HiSeq4000. The accessions belonging to *P. pyrifolia* include Amanogawa (AG), Niitaka (NK), Whangkeumbae (HKB), Youngsanbae (YS), Imamuraaki (KC), SuperGold (SG), Wonwhang (WH), and Chojuro (CJ). The two accessions of *P. ussuriensis* are DG and CDR, and those of *P. bretschneideri* are Yali (YL) and Dangshansuli (DSHS); the two accessions of *P. calleryana* are OPR125 and OPR195; Bartlett (BTL) and Max Red Bartlett (MRB) are the two types of European pears (*P. communis*) used, and Godang 5-1 (GD) is the *P. fauriei* variety used; lastly, Fuji (FJ) variety of the *M. domestica* taxon and the unknown species Kozo (KZ) are sequenced. The cp genome sequences of the 19 species were deposited in GenBank, and their accession numbers are listed in Table 1. Similar to other land plants, all the assembled cp genomes exhibit conserved circular DNA and quadripartite structures (Figure 1). The lengths of the genomes ranged from 159 kb (10 species) to 160 kb (9 species) (Table 1). The DG variety of *P. ussuriensis* has the smallest genome size (159,879 bp), while the HKB variety (160,153 bp) has the largest genome size. Each of the 19 genomes included four parts, namely, a large single-copy (LSC), a small single-copy (SSC), and a pair of inverted repeat (IR) regions (denoted as IRa and IRb). The length of each region is displayed in Table 1. The average GC content of all the genomes was ∼36.5%, which was nearly identical in all 19 genomes analyzed herein. The GC contents of the LSC, SSC, and IR regions were ∼34%, ∼30%, and 43%, respectively, and the higher GC contents observed in the IR regions were attributed to the presence of four GC-rich duplicated rRNA genes. In total, all the *Pyrus* and the *Malus* species showed similar sizes, nucleotide compositions, and region arrangements.

**TABLE 1:** Summary of the complete chloroplast genome statistics of 18 *Pyrus* and one *Malus* species.

	AG^a^	NK^a^	HKB^a^	YS^a^	KC^a^	SG^a^	WH^a^	CJ^a^	DG^b^	CDR^b^	YL^c^	DSHS^c^	OPR125^d^	OPR195^d^	GD^e^	KZ^f^	BTL^g^	MRB^g^	FJ^h^
Genome size (bp)	160,097	160,083	160,153	160,097	159,894	159,926	159,924	159,885	159,879	160,055	159,907	159,905	160,080	160,126	160,063	159,885	159,966	159,928	160,069
LSC size (bp)	88,111	88,066	88,164	88,111	87,868	87,898	87,896	87,863	87,858	88,069	87,880	87,877	88,058	88,099	88,052	87,863	87,893	87,855	88,184
SSC size (bp)	19,214	19,245	19,217	19,214	19,242	19,244	19,244	19,238	19,237	19,214	19,243	19,244	19,240	19,255	19,239	19,238	19,251	19,251	19,181
IR size (bp)	26,386	26,386	26,386	26,386	26,392	26,392	26,392	26,392	26,392	26,386	26,392	26,392	26,391	26,386	26,386	26,392	26,411	26,411	26,352
Number of genes	127	127	127	127	127	127	127	127	127	127	127	127	127	127	127	127	127	127	127
Protein-coding genes	83	83	83	83	83	83	83	83	83	83	83	83	83	83	83	83	83	83	83
tRNA genes	36	36	36	36	36	36	36	36	36	36	36	36	36	36	36	36	36	36	36
rRNA genes	8	8	8	8	8	8	8	8	8	8	8	8	8	8	8	8	8	8	8
Genes duplicated in IR	17	17	17	17	17	17	17	17	17	17	17	17	17	17	17	17	17	17	17
GC content (%)	36.56	36.56	36.55	36.56	36.57	36.57	36.57	36.58	36.57	36.57	36.57	36.57	36.58	36.56	36.57	36.57	36.56	36.57	36.55
GenBank accession	OK545532	KX904342	KX450877	OK545533	KX825882	KX825885	KX450876	OK545534	OK545535	OK545536	KX450881	KX450880	OK545537	OK545538	OK545529	OK574454	KX450879	OK545530	OK545531

AG, Amanogawa; NK, Niitaka; HKB, Whangkeumbae; YS, Youngsanbae; KC, Imamuraaki; SG, SuperGold; WH, Wonwhang; CJ, Chojuro; DG, Doonggeullebae; CDR, Cheongdangrori; YL, Yali; DSHS, Dangshansuli; GD, Godang 5-1; KZ, Kozo; BTL, Bartlett; MRB, Max Red Bartlett; FJ, Fuji.

^a^ *P. pyrifolia*

^b^ *P. ussuriensis*

^c^ *P. bretschneideri*

^d^ *P. calleryana*

^e^ *P. fauriei*

^f^ unknown species

^g^ *P. communis*

^h^ *M. domestica*

#### 2.1.2 Gene structure

The cp gene numbers typically range from 110 to 130 in land plants, particularly in fruit trees of the Rosaceae family (Daniell et al, 2016; Xue et al, 2019). In this study, all the *Pyrus* and *Malus* species had the same number of functional genes (127 genes), including 83 protein-coding, 36 tRNA, and 8 rRNA genes (Table 2; Supplementary Table S1). There were 17 duplicate genes, namely, *trnA-UGC*, *trnE-UUC*, *trnL-CAA*, *trnM-CAU*, *trnR-ACG*, *trnV-GAC*, *trnN-GUU*, *rps7*, *rps12*, *rpl2*, *rpl23*, *ndhB*, *ycf2*, *rrn16S*, *rrn23S*, *rrn4.5S*, and *rrn5S*, in the IR region, and two genes *trnE-UUC* and *trnM-CAU* in the LSC region (Table 2). Furthermore, 17 intron-containing genes were found in all the sequenced species, of which 15 genes (*trnA-UGC*, *trnE-UUC*, *trnK-UUU*, *trnL-UAA*, *trnS-CGA*, *rps12*, *rps16*, *rpl2*, *rpl22*, *rpoC*, *ndhA*, *ndhB*, *petB*, *petD*, and *atpF*) have one intron, while *ycf3* and *clpP* have two introns each (Supplementary Table S2).

**TABLE 2:** List of annotated genes in the 18 *Pyrus* species.

Category	Group of genes	Names of genes
Self-replication	Large subunit of ribosome proteins	*rpl2* ^a^ (2x), *14*, *16*, *20*, *22* ^a^, *23* (2x), *32*, *33*, *36*
	Small subunit of ribosome proteins	*rps3*, *4*, *7* (2x), *8*, *11*, *12* **(2x), *14*, *15*, *16* ^a^, *18*, *19*
	DNA-dependent RNA polymerase	*rpoA*, *B*, *C1* ^a^, *C2*
	rRNA	*rrn16S* (2x), *rrn23S* (2x), *rrn4.5S* (2x), *rrn5S* (2x)
	tRNA	*trnA-UGC* ^a^ (2x), *trnC-GCA*, *trnD-GUC* *trnE-UUC* ^a^ (2x) (1x), *trnF-GAA*, *trnG-GCC*, *trnH-GUG*, *trnK-UUU* ^a^, *trnL-CAA* (2x), *trnL-UAA* ^a^, *trnL-UAG*, *trnM-CAU* (2x) (2x) *trnN-GUU* (2x), *trnP-UGG*, *trnQ-UUG* *trnR-ACG* (2x), *trnR-UCU*, *trnS-CGA* ^a^ *trnS-GCU*, *trnS-GGA*, *trnS-UGA*, *trnT-GGU* *trnT-UGU*, trnV-GAC (2x), *trnW-CCA*, *trnY-GUA*
Photosynthesis	Photosystem I	*psaA*, *B*, *C*, *I*, *J*
	Photosystem II	*psbA*, *B*, *C*, *D*, *E*, *F*, *I*, *J*, *K*, *L*, *M*, *N*, *T*, *Z*
	NADH-dehydrogenase	*ndhA* ^a^, *B* ^a^ (2x), *C*, *D*, *E*, *F*, *G*, *H*, *I*, *J*, *K*
	Cytochrome b/f complex	*petA*, *B* ^a^, *D* ^a^, *G*, *L*, *N*
	ATP synthase	*atpA*, *B*, *E*, *F* ^a^, *H*, *I*
	Rubisco	*rbcL*
Other genes	Maturase	*matK*
	Protease	*clpP* **
	Envelop membrane protein	*cemA*
	Subunit of acetyl-CoA-carboxylase	*accD*
	C-type cytochrome synthesis gene	*ccsA*
Unknown function	Conserved open reading frames	*ycf1*, *2* (2x), *3* **, *4*

^a^ Genes containing one intron; ** genes containing two introns; (2x) alone indicates genes duplicated in the IR region with the following (1x) or (2x) indicating genes duplicated in the LSC region.

### 2.2 Chloroplast genome characterization

#### 2.2.1 Comparison of intron length

The one *Malus* and 18 *Pyru*s cp genomes possessed 17 common intron-containing genes (5 tRNA and 12 protein-coding genes). Five of these intron-containing genes located in the IR region, namely, *ndhB*, *rpl2*, *rps12*, t*rnA-UGC*, and *trnE-UUC*, as well as the *trnL-UAA* gene located in the LSC region, appear to have the same intron lengths as those found in the Pomoideae conserved sequences. Highly polymorphic cp genomes in the *Aster* species were shown to have the same intron lengths for two genes (*rpl2* and *trnA-UGC*) located in the IR region (Tyagi et al., 2020). We observed the same intron lengths in all pears with *trnK-UUU*, and the first introns of the *ycf3* and *rpoC1* genes as well as the second intron of the *ycf3* gene showed Asian pear conserved sequences. One *P. ussuriensis* accession (CDR) and the AG group have the same CDSs in the cp genome (Supplementary Table S2). AG is the mother plant of NK, while HKB and YS are the progenies of NK. We named the AG group with AG, NK, HKB, and YS accessions as they have the same matrilineage. Additionally, in the KC group, KC and SG have the same mother cp background. Two of the cp genome sequences of *P. bretschneideri* shared the same intron lengths as the KC group. The large size variations in the genes *clpP* (35 bp, first with 10 bp and second with 25 bp), *ndhA* (28 bp), *petB* (11 bp), *rpl22* (20 bp), and *rps16* (43 bp) could assist the insertion–deletion (InDel) polymorphism markers in the classification of *Pyrus* and Pomoideae. We designed the primers for the exon region and amplified these regions to obtain the polymorphic intron lengths. Our previous study on the development of cp-genome-based InDel markers using *clpP* and *ndhA* involved detection of these intron-length polymorphisms in agarose gel. We denote KZ as an unclassified species; from the intron-length analysis, it was found that the intron length of KZ was exactly similar to that of the DG and KC groups; hence, we propose that its cp inheritance may be from *P. ussuriensis* or *P. pyrifolia*. Itai and Fujita (2008) noted in their study of the CAPS analysis of 1-aminocyclopropane-1-carboxylate synthase genes that KZ may belong to *P. pyrifolia*. Comparisons of the intron lengths of these versatile species with similar species showed that they are assembled and annotated uniformly. Because the complete cp genome was assembled and annotated in the same pipeline, this helped to correctly classify and compare the sequences.

#### 2.2.2 Divergence analysis

The complete cp genome sequences of 18 *Pyrus* and one *Malus* species were compared using the mVISTA program (Supplementary Figure S1) and KC as the reference. The results clearly demonstrate that the genomes are highly conserved between the species. As reported previously in other genera (Alzahrani, 2021; Munyao et al, 2020) we observed that the conservation of the coding regions is greater than that of the non-coding regions. Additionally, the differences in the IR regions are less common than those of the single-copy regions. A comparison of the intron lengths in this study, in which five of the genes are located in the IR region, did not show variance in length (Table 3). The *ycf2* gene showed a 6-bp difference between the species, and it was the only gene modification identified in the exon region. The other most significant divergent regions include *rps16–trbQ, trnR–atpA, trnE–psbD, psbZ–trnG, trnT–trnL, ndhC–trnM, trnM–atpE, accD–psal, petB–petD, rps3–rpl22, ndhF–trnL*, and *ndhA* (Supplementary Figure S1). The genomes of *P. communis* and *M. domestica* showed markedly more variations compared to those of the other Asian pear varieties.

**TABLE 3:** Comparison of intron lengths in the chloroplast genomes of the 18 *Pyrus* and 1 *Malu*s species.

Gene		Location	CDR^d^, AG^c^ group	DG^d^, KC^c^ group	OPR125 ^f^	OPR195 ^f^	GD^g^	BTL^h^ group	FJ^i^	Length variance^b^
*atpF^a^*		LSC	731	732	731	731	731	734	732	3 bp
*clpP*	Intron 1	LSC	830	827	828	826	827	825	820	5 bp
	Intron 2		624	634	635	634	636	637	649	13 bp
*ndhA*		SSC	1,132	1,156	1,131	1,133	1,128	1,130	1,141	28 bp
*ndhB^a^*		IR	669	669	669	669	669	669	669	-
*petB*		LSC	788	798	798	798	799	799	797	11 bp
*petD^a^*		LSC	724	724	724	724	724	724	724	-
*rpl2^a^*		IR	686	686	686	686	686	686	686	-
*rpl22*		LSC	111	91	91	91	91	91	91	20 bp
rpoC1**		LSC	738	738	738	738	738	737	744	1 bp
*rps12^a^*		IR	540	540	540	540	540	540	540	-
*rps16*		LSC	880	905	882	883	884	896	862	25 bp
ycf3**	Intron 1	LSC	707	707	707	707	707	707	708	-
	Intron 2		743	743	744	743	743	745	744	2 bp
*trnA-UGC^a^*		IR	807	807	807	807	807	807	807	-
*trnE-UUC^a^*		IR	948	948	948	948	948	948	948	-
trnK-UUU**		LSC	2,491	2,491	2,491	2,491	2,491	2,496	2,515	5 bp
*trnL-UAA^a^*		LSC	516	516	516	516	516	516	516	-
*trnS-CGA*		LSC	681	681	679	680	680	681	677	2 bp

^a^ Including FJ, all intron-containing genes have the same intron length; ** except for European pear and FJ varieties, the intron-containing genes of Asian pears have the same intron length.

^b^ Length variance indicates the length variations between the *Pyrus* species; CDR: Cheongdangrori; AG group (AG, Amanogawa; NK, Niitaka; HKB, Whangkeumbae; YS, Youngsanbae); DG, Doonggeullebae; KC group (KC, Imamuraaki; SG, SuperGold; WH, Wonwhang; CJ, Chojuro; YL, Yali; DSHS, Dangshansuli); GD, Godang 5-1; KZ, Kozo; BTL group (BTL, Bartlett; MRB, Max Red Bartlett); FJ, Fuji.

^c^ *P. pyrifolia*

^d^ *P. ussuriensis*

^e^ *P. bretschneideri*

^f^ *P. calleryana*

^g^ *P. fauriei*

^h^ *P. communis*

^i^ *M. domestica*

**FIGURE 2:**
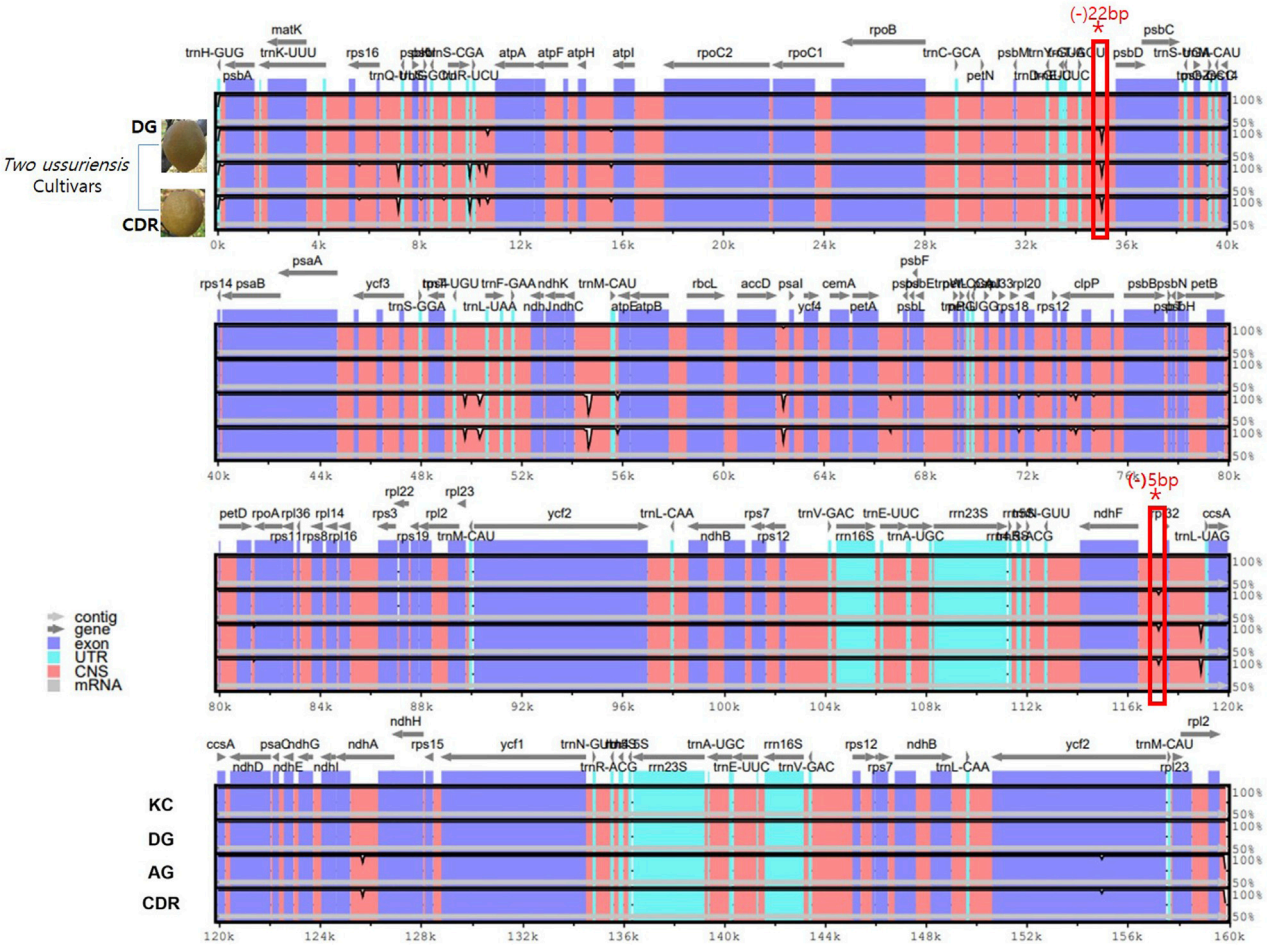
Divergence analysis using sequence alignment of four *Pyrus* cp genomes using mVISTA with KC as the reference. The vertical scale indicates the percentage of identity, which ranges from 50% to 100%. The horizontal axis indicates the coordinates within the cp genome. The genomic regions are color-coded. Two of the *P. ussuriensis* accessions are shown on lanes 2 and 4. The rectangular box highlighted in red color shows the detected deletion sequences for 22 bp in DG-*trnT-GGU*-*psbD* and 5 bp in DG-ndhF-rpl32 that are shared by DG and CDR.

To clarify the cp genome comparisons, four accessions were selected and analyzed using mVISTA with KC as the reference. Based on the sequence alignment, two *P. ussuriensis* accessions (CDR and DG) showed dynamic polymorphisms (Figure 2). Two deletion sequences with 22 bp in DG-*trnT-GGU*-*psbD* and 5 bp in DG-*ndhF*-*rpl32* were detected as the cp genome of the KC reference, whereas numerous polymorphisms are detected in DG and CDR. These divergent regions could be used as potential markers for identifying the accessions of both Asian and European pears.

#### 2.2.3 Application of InDel polymorphism markers in the classification of *Pyrus*

**FIGURE 3:**
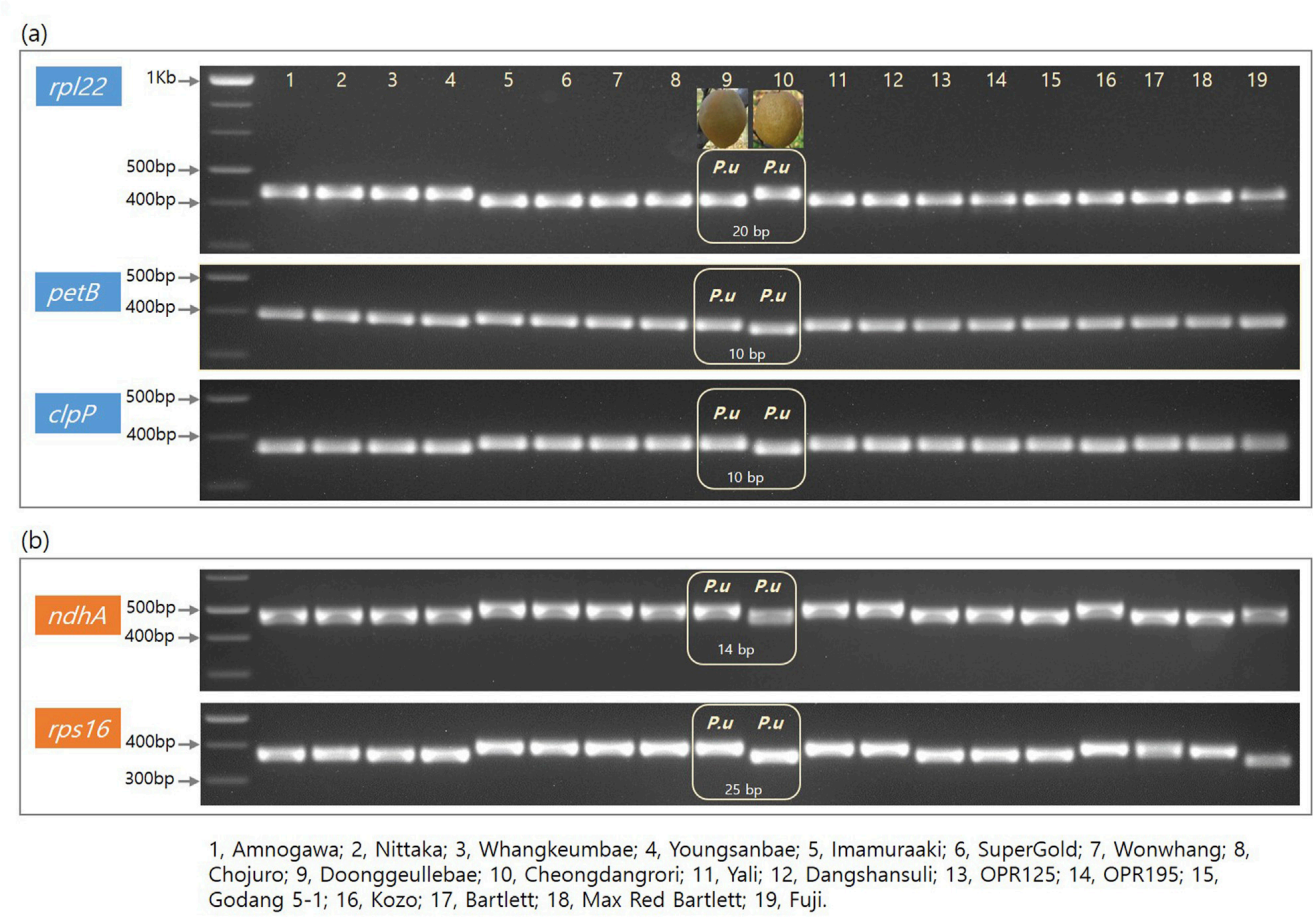
Electroporation photos for five genes from the cp genome of 18 *Pyru*s and one *Malus* species. Columns 1–19 are arranged in the following order: Amanogawa, Niitaka, Whangkeumbae, Youngsanbae, Imamuraaki, SuperGold, Wonwhang, Chojuro, Doonggeullebae, Cheongdangrori, Yali, Dangshansuli, OPR125, OPR195, Godang 5-1, Kozo, Bartlett, Max Red Bartlet, and Fuji. The gene names and size markers are shown along the left axis.

We designed five primers for the set of genes *rpl2, petB, clpP*, *ndhA*, and *rps16*. These amplicon products were adjusted to approximately 400 bp each (Table 4). The amplified polymorphic patterns were divided into two groups; the first mainly showed two different sizes between the (DK, KC) and (CDR, AG) groups (Figure 3A); the second involved two differentiations between these groups along with versatile polymorphisms in the speciation (Figure 3B). The *rpl22*, *petB*, and *clpP* genes in the CDR and AG groups were detected with deleted sequences, while the other genes did not show any polymorphism (Figure 3A). Thus, we can have shared polymorphisms in the *Pyrus* species before its speciation. Figure 3B shows that the *Pyrus* genome also has other InDels with speciation evolution.

**TABLE 4:** Primer information of the insertion–deletion polymorphism markers of the *Pyrus* species.

Gene name	Forward sequence	Reverse sequence	Target size
*rpl22*	ATC​GTG​AAA​CGT​GAC​ATC​TG	GCG​GTC​CTA​TGA​AGA​AAC​ACT	428–448 bp
*petB*	TCC​AAT​GGT​TCT​TAC​TCA​GGG​A	ACG​GCT​CAA​ACA​GAA​ACA​CC	399–410 bp
*clpP*	ACT​ATG​ATG​GCT​CCG​TTG​CT	ACG​TCT​AGC​ATT​CCC​TCA​CG	390–403 bp
*ndhA*	GTA​GGA​TGG​ATA​ACT​ATC​GGC	GTA​CTC​CCC​ATG​ACA​CGA​TT	486–514 bp
*rps16*	CGT​ACG​GCT​CGA​GAA​AAT​TC	CAC​CGA​AGT​AAT​GTC​TAA​ACC​CA	368–411 bp

#### 2.2.4 Phylogenetic analysis

**FIGURE 4:**
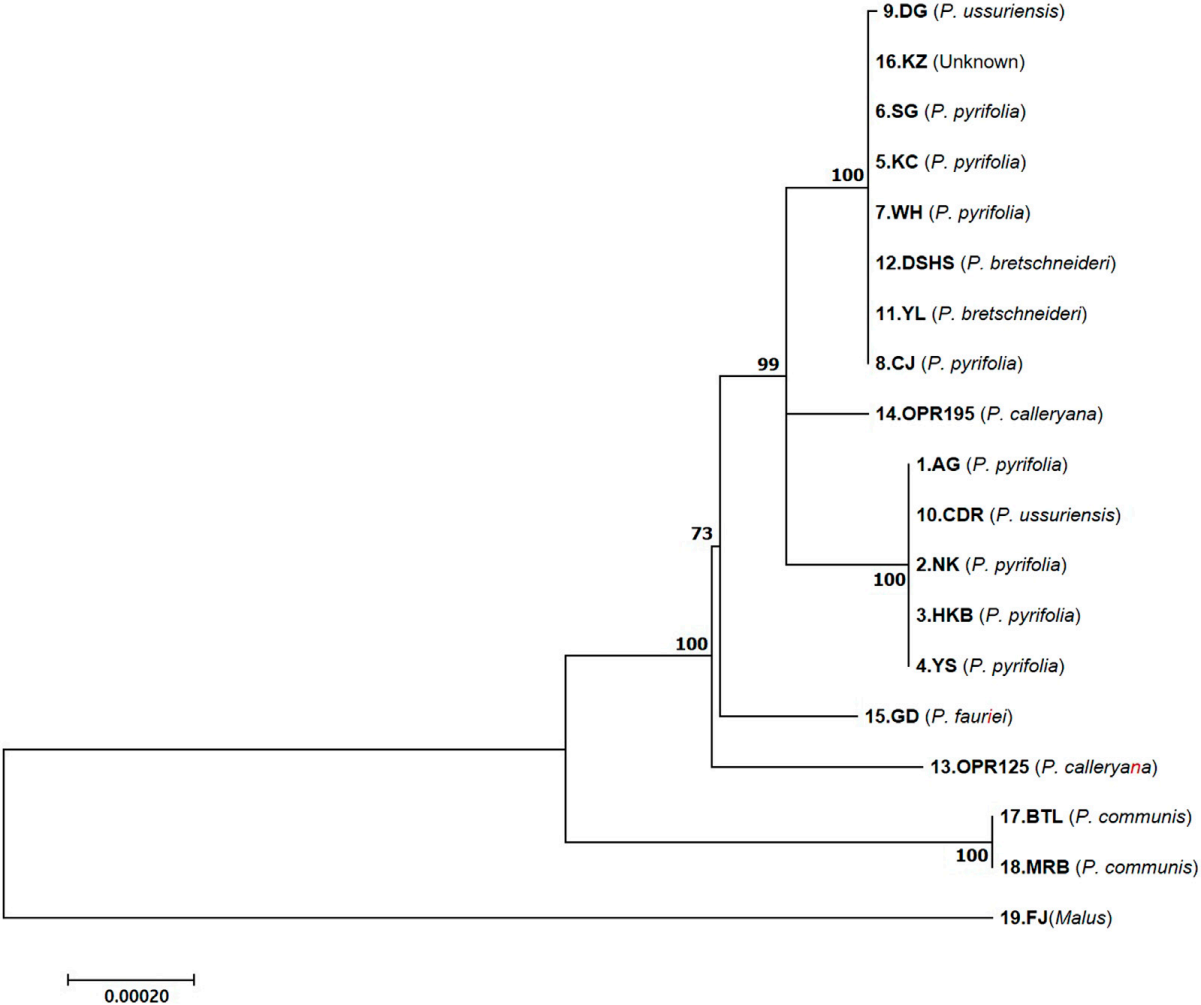
Maximum likelihood phylogenetic tree of the 18 *Pyrus* and one *Malus* species based on the coding sequences (CDSs) + introns of the different regions of the cp genomes. The bootstrap support values (>50%) from 1,000 replicates are indicated at the nodes. The numbers below and above the branch points represent the confidence levels of the relationships between the paired sequences, as determined by bootstrap statistical analysis. The tree is drawn to scale, with the branch lengths measured in terms of substitutions per site. DK, Doonggeullebae; KZ, Kozo; SG, SuperGold, KC, Imamuraaki; WH, Wonwhang; DSHS, Dangshansuli; YL, Yali; CJ, Chojuro; AG, Amanogawa; CDR, Cheongdangrori; NK, Niitaka; HKB, Whangkeumbae; YS, Youngsanbae; GD, Godang 5-1; BTL, Bartlett; MRB, Max Red Bartlett; FJ, Fuji.

Completed cp genomes, CDSs, and CDSs + introns of the LSC, SSC, and IR regions were used to construct a phylogenetic tree of the 18 *Pyrus* species, and the *Malus* species was used as the outgroup. The phylogenetic tree using the coding and intron sequences showed clustering of two groups (Figure 4). The first cluster comprised one *P. ussuriensis* (DG), four *P. pyrifolia* (KC, SG, WH, and CJ), two *P. bretschneideri* (YL and DSHS), and the unknown (KZ) species. The second cluster included one *P. ussuriensis* (CDR) and four *P. pyrifolia* (AG, YS, NK, and HKB) species. NK is the maternal species to YS and HKB, while AG is the maternal species to NK. Interestingly, two *P. bretschneideri* accessions (YL and DSHS) were also grouped in the DG and KC branches, even though these accessions are mainly cultivated in Mainland China. The OPR125 and OPR195 accessions of *P. calleryana* as well as GD accession of *P. fauriei* were found to be parental nodes in different branches. From this phylogenetic tree, it is clear that the two accession groups of Asian pears (AG and KC) originated from GD (*P. fauriei*). These results are consistent with the complete phylogenetic tree constructed using CDSs (Supplementary Figure S1A) and complete genomes (Supplementary Figure S1B); furthermore, the three different phylogenic trees are the same without GD and the OPR125 node when using cp CDSs.

## 3 Discussion

Recent research on the Rosaceae family has achieved significant strides in understanding its evolutionary dynamics and genetic diversity through cp genome analysis. A recently reported study involved sequencing and comparison of the cp genomes of several species within the *Rubus* genus. Eight newly assembled cp genomes were compared, and the most diverse regions were found to be the intergenic spacers, including *rps16*–*trnQ*, *trnL*–*trnT*, and *rpl32*–*trnL*–*ccsA* (Yu et al., 2022). In addition, comparative cp genomics of the *Rubus* family showed that there were significant differences in the lengths and positioning of four genes: *rbcL* and *psaB* are involved in photosynthesis, *matK* is associated with cp class II intron splicing, and *rpl32* is related to protein synthesis. The authors suggested that these genes may have experienced special evolutionary history (Lu et al., 2024). Comparison of the entire cp genome sequence of the Qixiadaxiangshui pear showed several mutation hotspots with precise Pi values, such as *ndhC*-*trnM-CAU* and *trnR-UCU*-*atpA*. These regions are known to undergo faster nucleotide substitutions at the species level, providing important references for the development of DNA barcodes (Jiao et al., 2024). We report that the other most significant divergent regions include *rps16–trbQ, trnR–atpA, trnE–psbD, psbZ–trnG, trnT–trnL, ndhC–trnM, trnM–atpE, accD–psal, petB–petD, rps3–rpl22, ndhF–trnL*, and *ndhA* (Supplementary Figure S1). Almost all of the cp genome comparative analysis regions show codon usage changes and microsatellites. The current study is limited by the sample size and versatile species used; however, we expanded the study with seven species of pear and newly assembled their pipeline along with comparative analysis.

The analysis was confined to the cp genome, which represents only the maternal lineage. A more comprehensive understanding of evolutionary history would require additional research incorporating nuclear and mitochondrial genomes. The cp analysis of various species was aimed at examining these correlations along with compilation of the largest genetic resources. Reconstruction of the largest pedigree network for pear accessions and evaluation of the genetic diversity of the USDA-ARS national *Pyrus* collection study was published in 2020. This involved the Applied Biosystems Axiom Pear 70 K Genotyping Array and allowed high-density single-nucleotide polymorphism (SNP)-based genotyping of almost the entire collection. A total of 1,890 diploid *Pyrus* spp. samples, two haploids, and five intergenic hybrids (x*Pyronia* = *Pyrus* × *Cydonia*; x*Sobopyrus* = *Sorbus* × *Pyrus*) were found. Removal of the duplicates resulted in 1,331 unique genotypes. After filtering for missing data and Mendelian errors, a total of 62,673 SNPs remained (Montanari et al., 2020). We divided the two *P. ussuriensis* accessions as types I and II. Here, type I was mainly composed of *P. pyrifolia* and *P. pyrifolia/ussuriensis* hybrid (*P. hybrid*) accessions, which are the CDR and AG groups in this study. Type II were mostly clustered along the border regions and composed of *P. calleryana*, *P. hybrid*, *P. communis pyraster*, *P. communis caucasica*, *P. pyrifolia*, and *P. communis* accessions (Supplementary Figure S2).

*De novo* genome assembly of the wild pear (*P. betulaefolia*) is widely used as the rootstock. Dong et al. (2020) used a Venn diagram to obtain the shared and unique gene families among the *P. betulaefolia, P. bretschneideri*, and *P. communis* species*.* Orthologous clustering of these three sequenced pear genomes showed 22,658 gene families, which was far greater than those in the DSHS and Bartlett genomes in the phylogenetic analysis. However, *P. betulaefolia* and *P. communis* had 3,677 common gene families containing 9,272 common genes, which was larger than that between *P. betulaefolia* and *P. bretschneideri* that had 2,390 common gene families containing 5,629 common genes (Dong et al., 2020 *)*. There is no reproductive isolation in pear plants, which results in widespread interspecific hybridization. We seek to understand the evolution of the pear genome and construct its parentage clearly, which are limited by genome sequences of the different plant species and hybrid breeding technologies that affect the exact parentage history even with natural hybridization. Our InDel markers are amplified in the *Malus* species. Our study can therefore be expanded to the entire *Pyrus* germplasm collection to assist with the evaluation of the maternal heritage.

**FIGURE 5:**
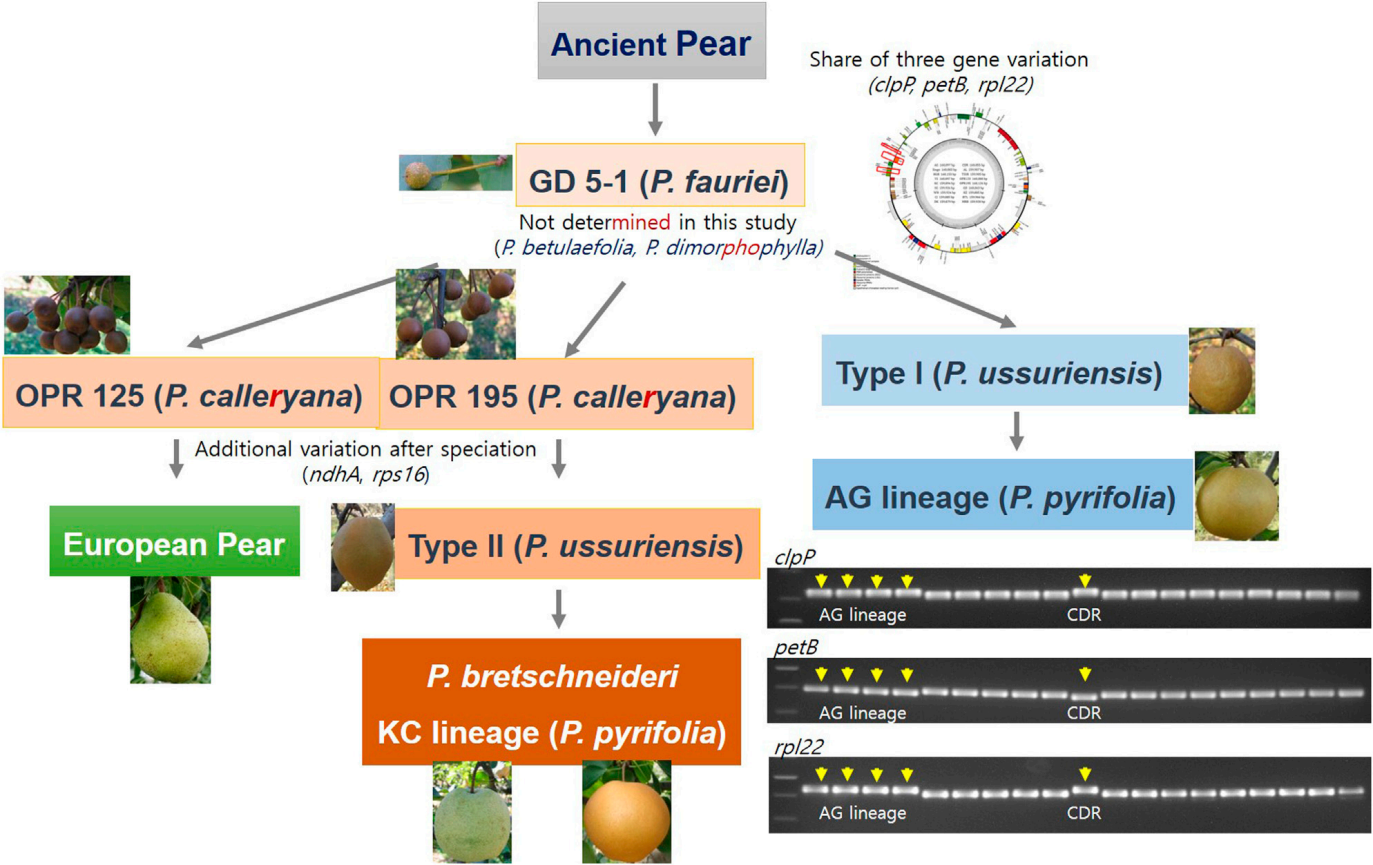
Evolutionary model of the versatile *Pyrus* species from the ancient pear variety. Five insertion–deletion markers were found in this study based on the analysis of 18 *Pyrus* and one *Malus* species.

The evolution of the cp sequences is relatively slow and highly conserved between species within a genus (Dong et al., 2012). We utilized the InDel sequences and intron-length polymorphisms within the cp genomes to analyze the evolutionary relationships within the Rosaceae family and especially the *Pyrus* species. Based on our complete cp genome study, phylogenetic tree analysis, and development of five InDel markers and their applications, we suggest the pear evolution history (Figure 5). However, we did not study the *P. betulaefolia* and *P. dimorphophylla* accessions. We suggest that the three genes *rpl22*, *petB*, and *clpP* had deletion sequences in *Pyrus* in the LSC region neighboring the IRb region. The DG accession of *P. ussuriensis* forms the background of the AG group. The OPR195 and OPR125 wild pear accessions are derived from *P. fauriei* after undergoing three gene deletions each; there are also additional variations after speciation with the *ndhA* and *rps16* gene*s*. Type II *P. ussuriensis* comprises the DK cultivar, which is the ancestor of the KC cultivar and *P. bretschneideri*. The European pear is supposedly derived from the OPR125 variety of *P. calleryana* (Figure 5). The phylogenetic tree constructed using the complete cp genomes of the Rosaceae family had three branches of clustered species, with the Qixiadaxiangshui pear being clustered with the plants of the genera *Crataegus* and *Malus* on the same branch. The close clustering of the Qixiadaxiangshui (*P. bretschneideri*) pear with DSHS (*P. bretschneideri*), WH (*P. pyrifolia*), and YL (*P. bretschneideri*) suggests that these cultivars share a recent common ancestor (Jiao et al., 2024).

Interestingly, two of the *P. ussuriensis* accessions have different fruit shapes; DK (type II) is ovate while CDR (type I) is round (Figure 5). One of the *P. bretschneideri* varieties had pyriform shape. Quantitative trait loci (QTL) analysis was carried out for HKB (*P. pyrifolia*, round) × YL (*P. bretschneideri*, pyriform) and high-resolution melting (HRM) markers were developed. The *P. bretschneideri* fruit shapes are versatile for the white pear (TSSR, round) and YL (pyriform).

We reported the InDel polymorphisms in the *ndhA* and *clpP* genes in 18 accessions of the *Pyrus* species. We used the findings from our previous study for comparisons and conducted complete cp genome sequencing as well as gene annotation. The plant genetics point toward good taste, color, and consumer requirement characteristics even for the intercross accessions obtained from breeding. Five InDel markers were determined in this study, which could assist in elucidating the maternal origins of the Asian and wild pear varieties. The results of this study can be used in the follow-up of other pear genetic resources to clarify pear genome evolution. It may also be necessary to integrate other types of molecular marker in the future for analyzing more recent evolutionary events.

## 4 Materials and methods

### 4.1 Plant materials

We used eight *P. pyrifolia* accessions (NK, HKB, AG, YS, WH, KC, SG, and CJ), an unknown accession (KZ), two *P. ussuriensis* accessions (DG and CDR), two *P. bretschneideri* accessions (YL and DSHS), one *P. fauriei* accession (GD), two European pear accessions (BTL and MRB), and a classified rootstock group with two *P. calleryana* accessions (OPR125 and OPR195) in this study. Additionally, the FJ accession of *M. domestica* was included in the sampling as an outgroup. The plant samples were collected from Naju (latitude: 35° 01′25.6″N, longitude: 126° 44′38.4″E), the Pear Research Station, NIHHS, RDA, Republic of Korea. Plastid genomic DNA was extracted from young leaves using a DNeasy Plant Mini kit (Qiagen, CA, United States of America) according to manufacturer instructions.

### 4.2 Chloroplast genome sequencing, assembly, and annotation

Whole-genome sequencing was performed using an Illumina genome analyzer (HiSeq4000, Illumina, United States of America) platform at Macrogen (http://www.macrogen.com/), Seoul, Republic of Korea. Genomic libraries with a 350 bp insert size were prepared using the paired-end standard protocol recommended by the manufacturer, and each sample was tagged separately with a different index. NGS was performed to obtain 25–30 Gb targets, and the total read bases (bp) were arranged from 24,000,528,290 (CJ) to 30,796,318,040 (MRB). The raw sequencing data were trimmed using the quality trim program in QIAGEN CLC Assembly Cell package version 4.2.1 (QIAGEN Digital Insights, Denmark) and used for *de novo* assembly of the cp genomes according to a previous study (Kim et al. 2015). In brief, trimmed high-quality reads were *de novo* assembled using the clc_novo_assemble program in QIAGEN CLC Assembly Cell, and the cp contigs were then selected and ordered through similarity searches with reported *Pyrus* cp genomes (MK488091.1, MK172841.1, MF521826.1, and LT996903.1). The selected contigs were merged, and their gaps were filled to generate the final complete cp genomes. The errors were corrected through read mapping and manual curation. The complete cp sequence was annotated using the GeSeq (Tillich et al, 2017) and Artemis (Carver et al, 2012) programs with reported *Pyrus* cp genomes. In addition, the precise gene spans were determined by manual curation using BLAST searches. Genes with CDS lengths different from those of other cp genomes were subjected to manual curation through comparisons with the other cp genomes. The circular cp genome map of the 18 *Pyrus* species was drawn using OGDRAW (Lohse et al., 2007).

### 4.3 Divergence analysis

The complete cp genome sequences of the 18 *Pyrus* and one *Malus* species were compared using mVISTA with LAGAN mode from the VISTA suite of tools (Mayor et al., 2000). The annotation of the KC variety was used as the reference. Pairwise alignment of the DNA sequences was performed with mVISTA and the AVID alignment algorithm; the mVISTA visualization module was used to display the global sequence alignment, with the genes displayed on top along with nucleotide variations of the base sequences.

### 4.4 InDel marker polymerase chain reaction (PCR) and electrophoresis

PCR analysis was performed in accordance with the procedures of Chung et al. (2019). The amplified fragments were electrophoresed with 2.5% agarose gels (Sigma, A0169, United States of America). The size markers were followed by the manufacturer’s 1 kb DNA ladder marker (Enzynomics, DM003, South Korea).

### 4.5 Phylogenetic analysis

The CDSs of 79 conserved protein-coding genes were extracted, and concatenated sequences were generated for each of the 19 cp genome sequences by combining their CDSs. In addition, the CDSs with introns of 78 conserved protein-coding genes (excluding the *rps12* gene) were extracted, and concatenated sequences were generated for each of the 19 cp genome sequences. The concatenated sequences of CDSs and CDSs with introns were multiple aligned using the MAFFT program version 7 (https://mafft.cbrc.jp/alignment/server/index.html). (Katoh et al, 2019) Phylogenetic analyses were then performed on the aligned sequences using MEGA 7.0 (Kumar et al, 2016) with the maximum likelihood (ML) statistical method, general time reversible (GTR) substitution model, and bootstrapping with 1,000 replicates. For topological comparisons of the tree, whole cp genome sequences were used in the phylogenetic analyses.

## Data Availability

The datasets presented in this study can be found in online repositories. The names of the repositories and accession numbers can be found below: https://www.ncbi.nlm.nih.gov/, OK545532; https://www.ncbi.nlm.nih.gov/, KX904342; https://www.ncbi.nlm.nih.gov/, KX450877; https://www.ncbi.nlm.nih.gov/, OK545533; https://www.ncbi.nlm.nih.gov/, KX825882; https://www.ncbi.nlm.nih.gov/, KX825885; https://www.ncbi.nlm.nih.gov/, KX450876; https://www.ncbi.nlm.nih.gov/, OK545334; https://www.ncbi.nlm.nih.gov/, OK545335; https://www.ncbi.nlm.nih.gov/, OK545336; https://www.ncbi.nlm.nih.gov/, KX450881; https://www.ncbi.nlm.nih.gov/, KX450880; https://www.ncbi.nlm.nih.gov/, OK545337; https://www.ncbi.nlm.nih.gov/, OK545337; https://www.ncbi.nlm.nih.gov/, OK545229; https://www.ncbi.nlm.nih.gov/, OK574454; https://www.ncbi.nlm.nih.gov/, KX450879; https://www.ncbi.nlm.nih.gov/, OK545530; https://www.ncbi.nlm.nih.gov/, OK545531.

## Supplementary material

SUPPLEMENTARY FIGURE S1 Divergence analysis: sequence alignment of 1 *Malus* and 18 *Pyrus* chloroplast genomes by mVISTA, with KC as the reference. The vertical axis indicates the percentage of identity in the range of 50%–100%. The horizontal axis indicates the coordinates within the chloroplast genome. The genomic regions are color-coded.

SUPPLEMENTARY FIGURE S2 Pedigree network for all the trios and duos identified in this study. Each dot represents an accession, and species-based color-coding is used, as shown in the legend on the right-hand side. Relationships are shown with arrows from the parent to offspring accession. The blue box is type I *P. ussuriensis*, and the orange box is type II *P. ussuriensis*.

SUPPLEMENTARY FIGURE S3 Maximum likelihood phylogenetic tree of the 18 *Pyrus* and 1 *Malus* species based on the **(A)** coding sequence regions and **(B)** complete chloroplast genomes. The bootstrap support values (>50%) from 1,000 replicates are indicated at the nodes.
