# Supplementary figures and images for: Chloroplast genome sequencing and divergence analysis of 18 Pyrus species: insights into intron length polymorphisms and evolutionary processes
Source: Front Genet. 2024 Oct 23;15:1468596. doi: 10.3389/fgene.2024.1468596 (PMC11537901; doi:10.3389/fgene.2024.1468596)

Figure S2.

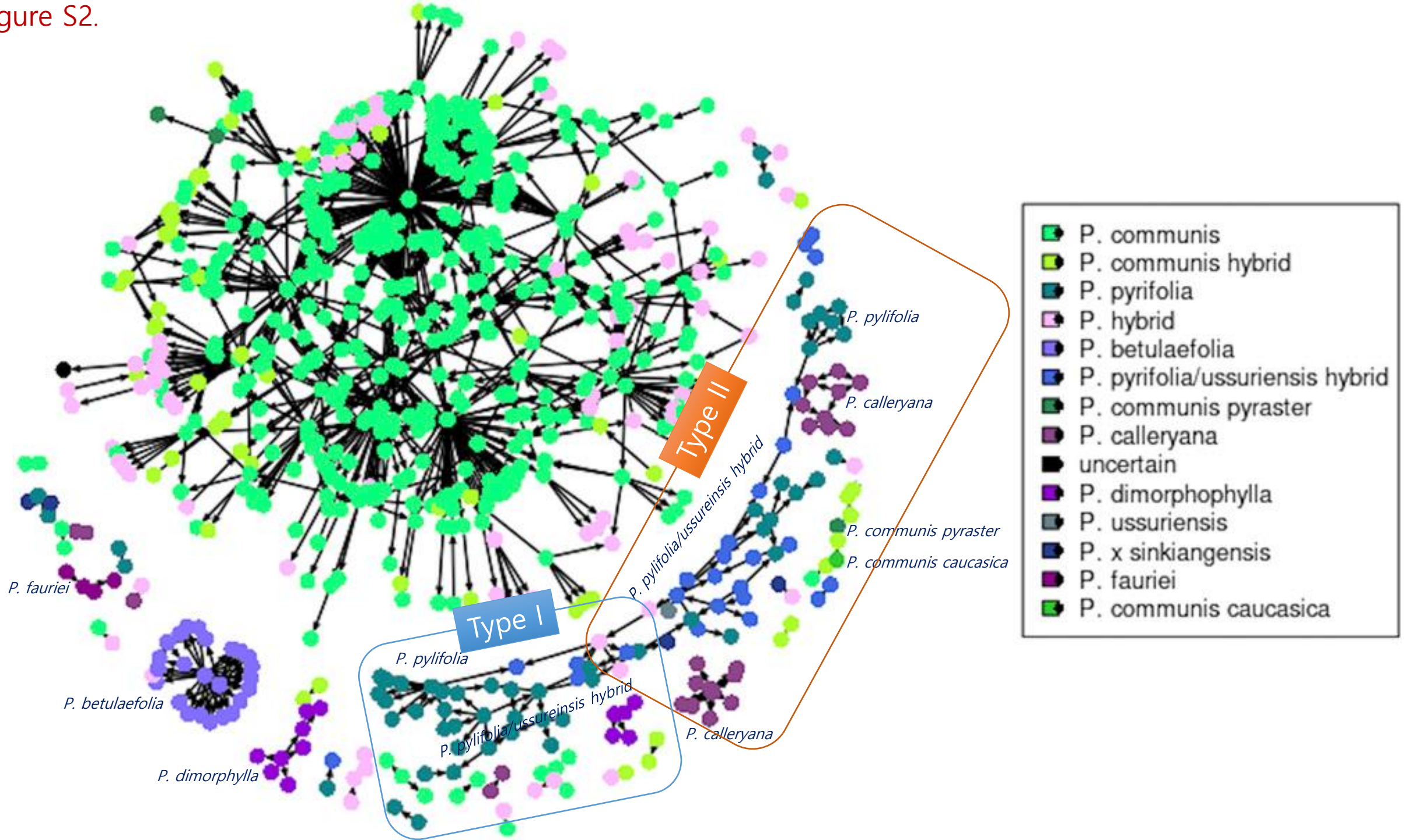

Supplement: Supplementary file 1 [file Image2.pdf]

Figure S3a.

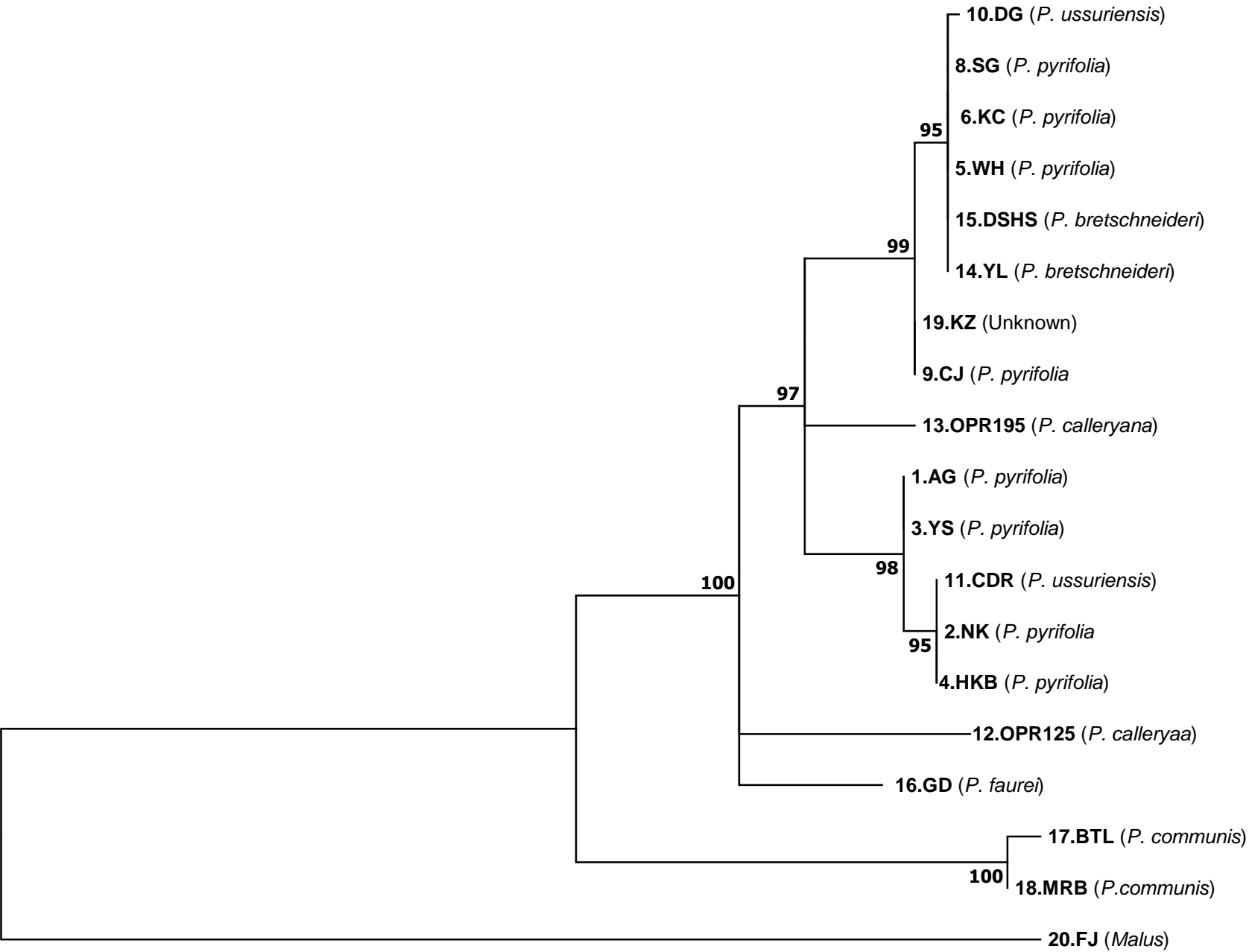

0.00020

Figure S3b.

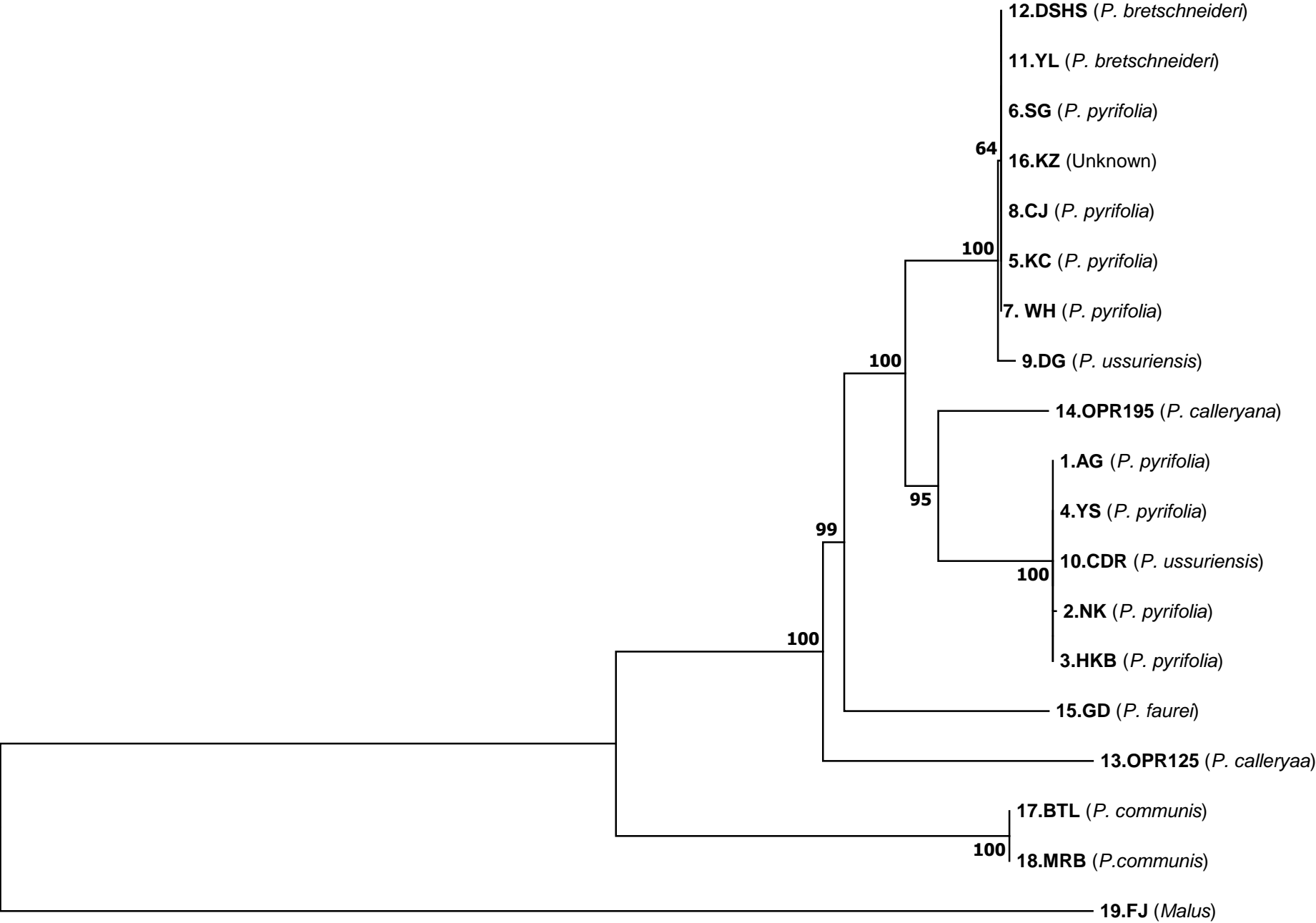

0.00050

Supplement: Supplementary file 2 [file Image3.pdf]

Figure S1.

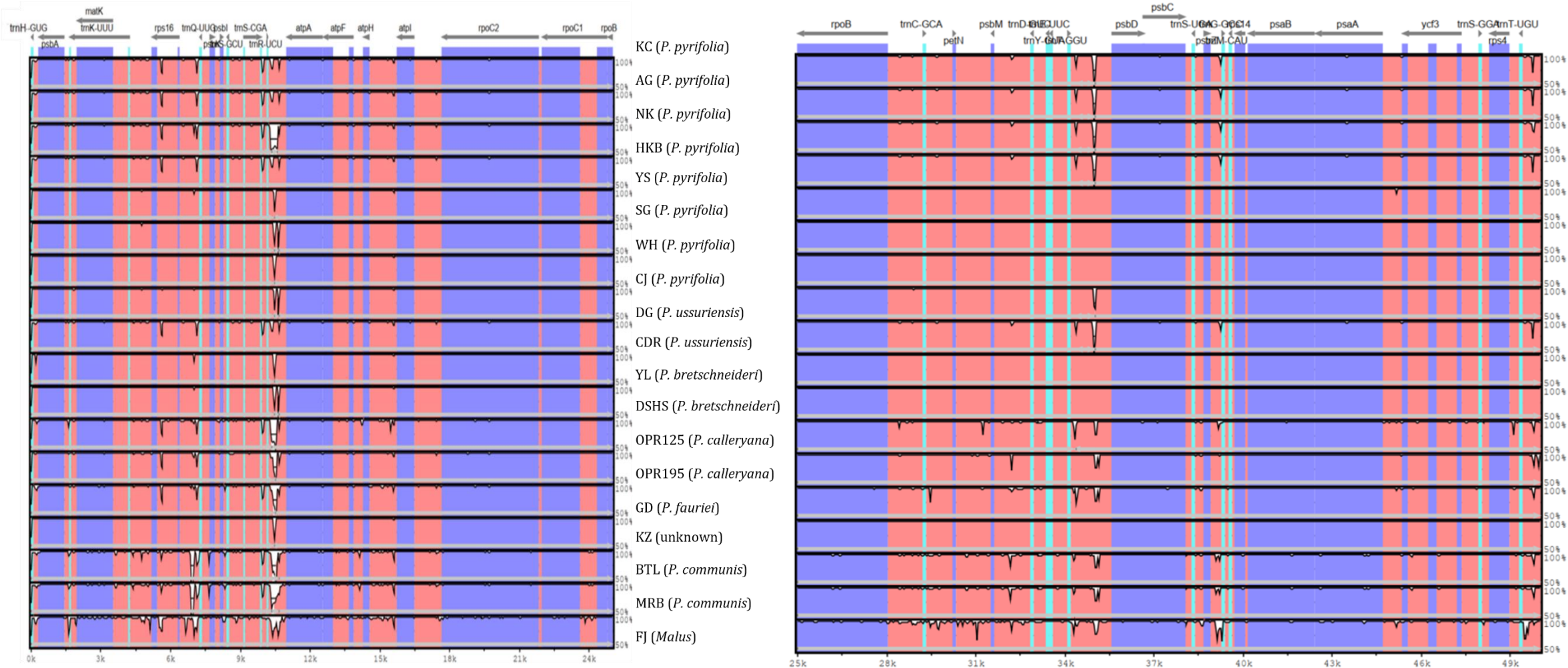

Figure S1.\_continued

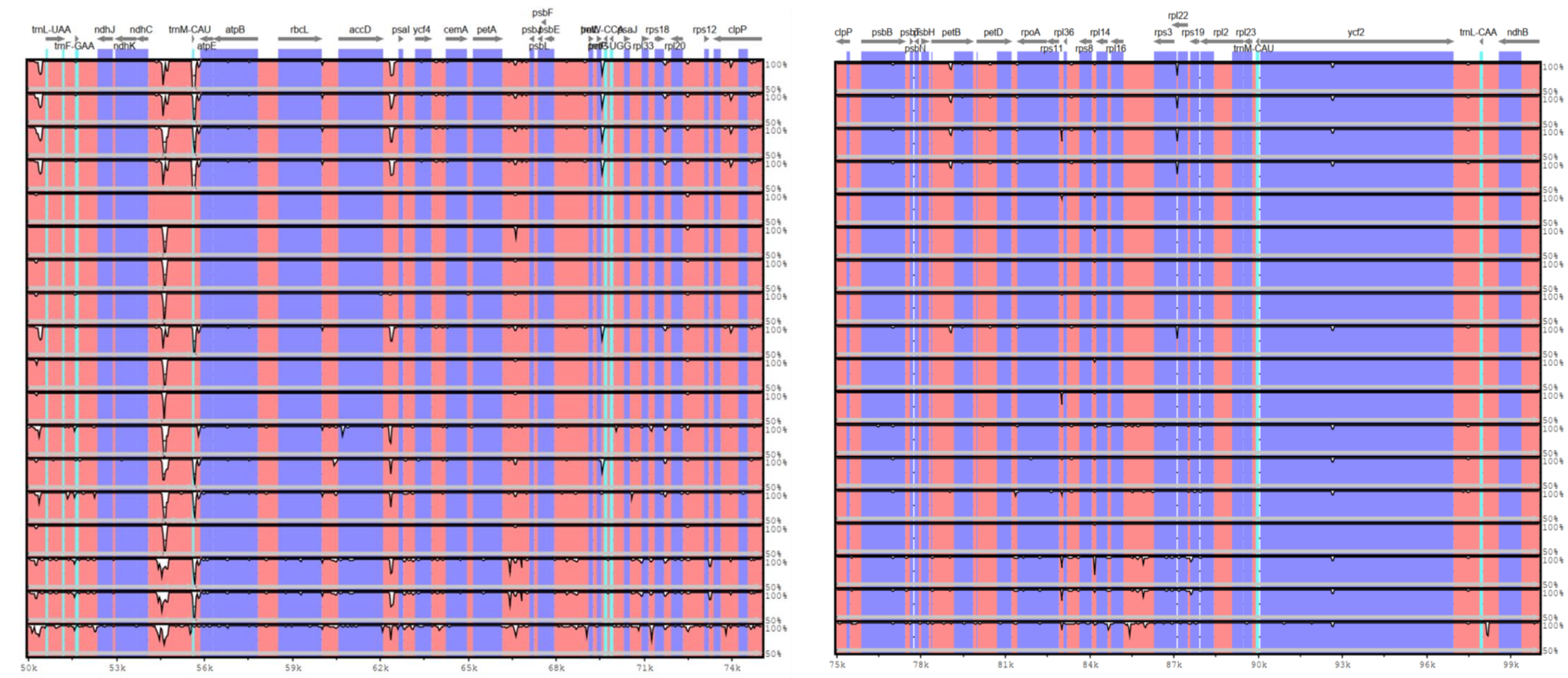

Figure S1.\_continued

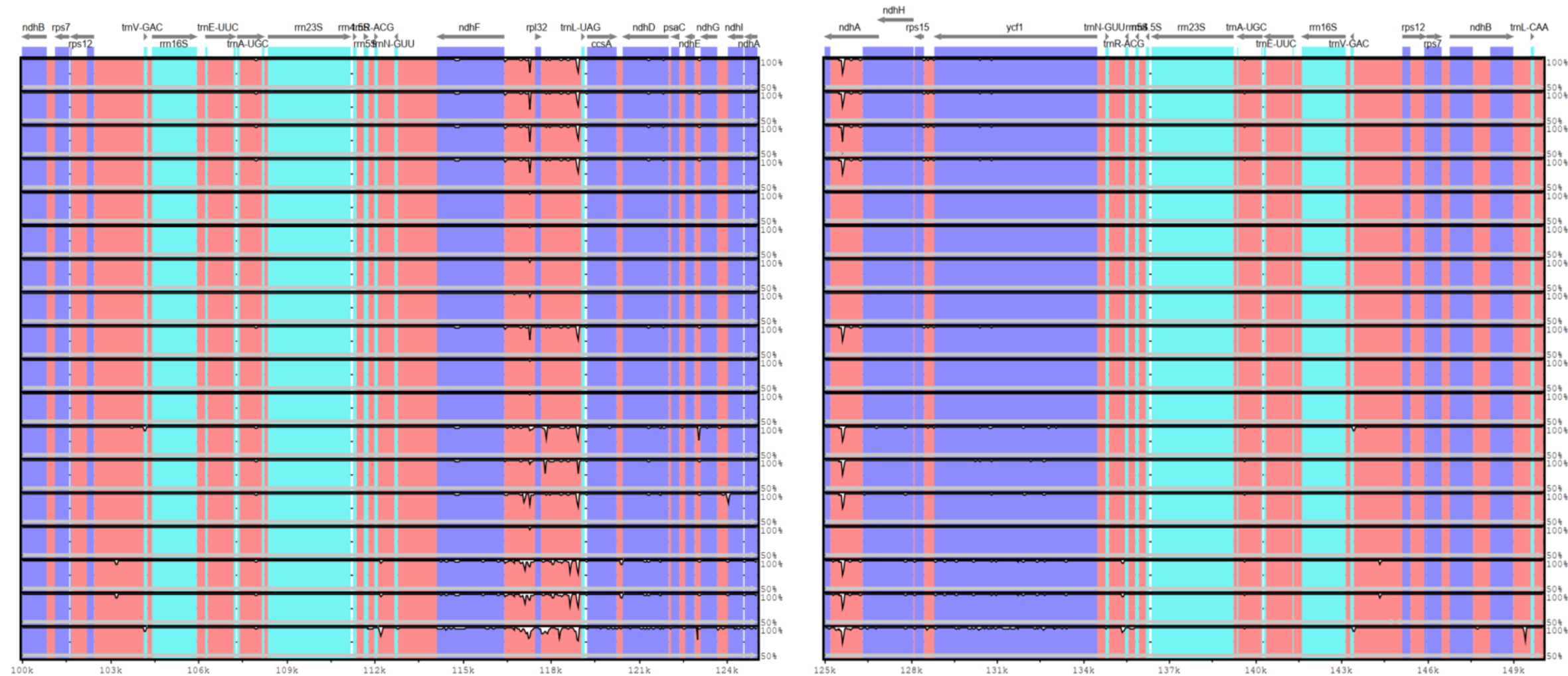

Figure S1.\_continued

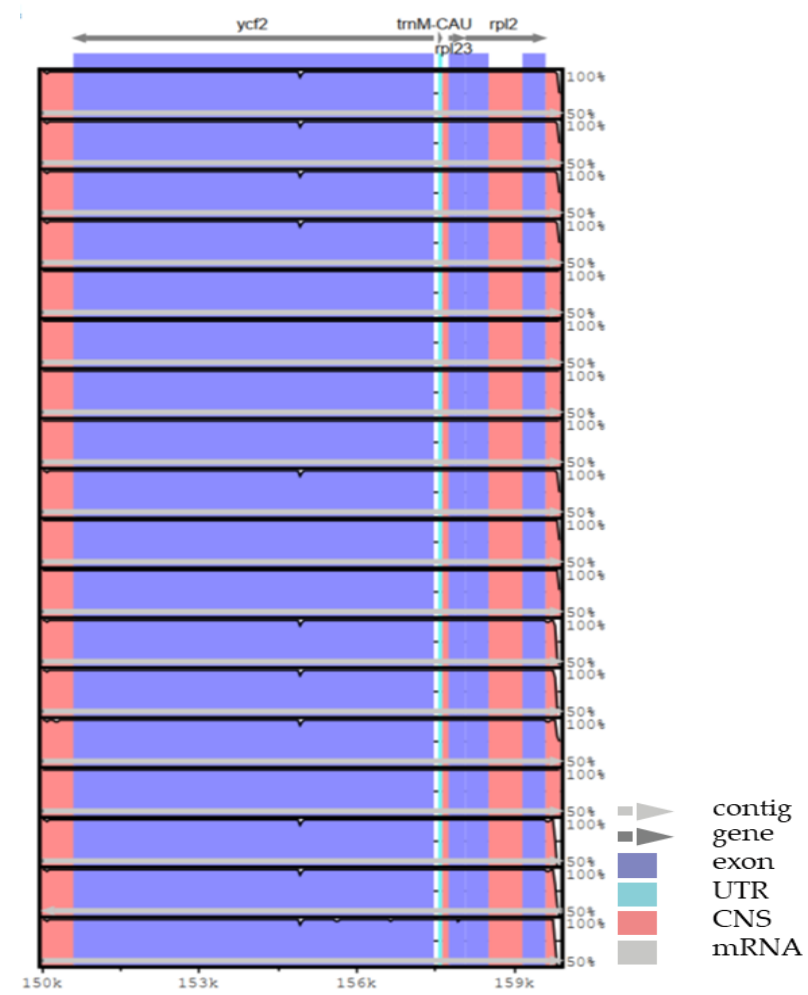

Supplement: Supplementary file 3 [file Image1.pdf]
